# An integrated health delivery platform, targeting soil-transmitted helminths (STH) and canine mediated human rabies, results in cost savings and increased breadth of treatment for STH in remote communities in Tanzania

**DOI:** 10.1186/s12889-019-7737-6

**Published:** 2019-10-28

**Authors:** Felix Lankester, Alicia Davis, Safari Kinung’hi, Jonathan Yoder, Catherine Bunga, Shayo Alkara, Imam Mzimbiri, Sarah Cleaveland, Guy H. Palmer

**Affiliations:** 1 Paul G. Allen School for Global Animal Health, Washington State University, Pullman, Washington USA; 2 Global Animal Health Tanzania, P.O. Box 1642, Arusha, Tanzania; 3 School of Geographical and Earth Sciences, University of Glasgow, Glasgow, UK; 4 National Institute of Medical Research, Mwanza, Tanzania; 5 School of Economic Sciences, Washington State University, Pullman, Washington USA; 6 Boyd Orr Centre for Population and Ecosystem Health, Institute for Biodiversity, Animal Health and Comparative Medicine, College of Medical, Veterinary and Life Sciences, University of Glasgow, Glasgow, UK

**Keywords:** Neglected tropical disease, Soil transmitted helminths, Rabies, One health, Integrated mass drug delivery, Tanzania

## Abstract

**Background:**

Achieving the Sustainable Development Goal of a 90% reduction in neglected tropical diseases (NTDs) by 2030 requires innovative control strategies.

This proof-of-concept study examined the effectiveness of integrating control programs for two NTDs: mass drug administration (MDA) for soil-transmitted helminths in humans and mass dog rabies vaccination (MDRV).

**Methods:**

The study was carried out in 24 Tanzanian villages. The primary goal was to demonstrate the feasibility of integrating community-wide MDA for STH and MDRV for rabies. The objectives were to investigate the popularity, participation and cost and time savings of integrated delivery, and to investigate the reach of the MDA with respect to primary school-aged children and other community members. To implement, we randomly allocated villages for delivery of MDA and MDRV (Arm A), MDA only (Arm B) or MDRV only (Arm C).

**Results:**

Community support for the integrated deliver**y** was strong **(**e.g**.** 85% of focus group discussions concluded that it would result in people getting “two for one” health treatments). A high proportion of households participated in the integrated Arm A events (81.7% MDA, 80.4% MDRV), and these proportions were similar to those in Arms B and C. These findings suggest that coverage might not be reduced when interventions are integrated. Moreover, in addition to time savings, integrated delivery resulted in a 33% lower cost per deworming dose and a 16% lower cost per rabies vaccination.

The median percentage of enrolled primary school children treated by this study was 76%. However, because 37% of the primary school aged children that received deworming treatment were not enrolled in school, we hypothesize that the employed strategy could reach more school-aged children than would be reached through a solely school-based delivery strategy.

**Conclusions:**

Integrated delivery platforms for health interventions can be feasible, popular, cost and time saving. The insights gained could be applicable in areas of sub-Saharan Africa that are remote or underserved by health services. These results indicate the utility of integrated One Health delivery platforms and suggest an important role in the global campaign to reduce the burden of NTDs, especially in hard-to-reach communities.

**Trial registration:**

clinicaltrials.gov NCT03667079, retrospectively registered 11th September 2018.

## Introduction

Almost a quarter of the world’s human population suffer the effects of 20 recognised neglected tropical diseases (NTDs) [1–5]. Despite the considerable investment that followed the Sustainable Development Goal of a 90% reduction in NTDs by 2030, the control of NTDs, which are poverty-promoting and occur primarily in rural areas of low-income countries, remains a profound health challenge [5, 6]. Novel, cost-effective, complementary and far-reaching control strategies are required to address this challenge.

The One Health concept recognises that the health of humans, animals and the environment are linked and that a multidisciplinary approach is required to address complex health problems [7, 8]. Given the role that all three factors play in the epidemiology of many NTDs, control efforts guided by the One Health approach has merit.

This study focuses on two NTDs endemic in East Africa: soil transmitted helminths (STH) and canine rabies. STH, a group of parasitic worms (including roundworms (*Ascaris lumbricoides)*, whipworms (*Trichuris trichuria*) and hookworms (*Necator americanus, Ancylostoma duodenale*)), infect more than 1.5 billion people in tropical and sub-tropical countries [9]. Where sanitation is poor, eggs present in human faeces contaminate the soil, exposing people, especially children, to infection through egg ingestion via dirty hands, contaminated water or food, and through the skin, especially while walking barefoot. Whilst STH can be treated, untreated cases are associated with pregnancy complications, anaemia, malnutrition and impaired early childhood physical and cognitive development and susceptibility to other diseases [10–14]. Fincham et al [13] and Le Hesran et al [14] Sparse data limits accurate prevalence estimates of STH across East Africa, however *N. americanus* is the most widely distributed species with other species having more restricted distributions [15].

In response to the World Health Organization’s (WHO) goal of treating more than 75% of school aged and pre-school aged children in endemic areas (an estimated 651.5 million) by 2020 [16], school-based mass drug administration (MDA) programs have been implemented as a cost-effective method of addressing the population group that bears the greatest burden of morbidity. In the United Republic of Tanzania, the 2017 national coverage of school-aged children receiving preventive chemotherapy for STH through the national primary school based deworming program (NSDP) was 90% [17]. In remote regions however, school-based programs can miss a significant proportion of children due to low attendance [18]. Additionally, some STH infections, such as hook worm, can be prevalent in adult populations. Therefore, if local elimination of STH is the goal then treatment of all age groups will be required [19].

Canine mediated human rabies is an NTD [5] responsible for 59,000 deaths and $8.6 billion in economic losses annually with rural Africa having the highest incidence (3.6 cases per 100,000) [20, 21]. Figures such as these have led the WHO, the Food & Agricultural Organization (FAO) and the World Organization for Animal Health (OIE) to recognize rabies as a global health priority and have committed to its elimination by 2030 (‘Zero by 30’) [22]. For this commitment to succeed, domestic dog vaccine delivery must be expanded from local to regional scales [23]. One barrier is the difficulty of consistently achieving the required coverage of 70% of the dog population across the hard-to-reach landscapes that characterize much of sub-Saharan Africa and Asia [24–26]. Where vaccination strategies rely on delivery through annual campaigns, low turnout in a few communities can jeopardise the success of the wider programme. Furthermore, team-led mass dog rabies vaccination (MDRV) campaigns are expensive, with costs up to $6.36 per dog vaccinated [26, 27].

The integration of preventative NTD programs with similar strategic approaches offers opportunities for financial and personnel cost savings, as well as improved program effectiveness through the wider adoption of integration strategies [28]. There are examples of integrated human health delivery strategies, for example the Schistosomiasis Control Initiative has included drugs targeting STH to its praziquantel regimen and the African Program for Onchocerciasis Control has provided an entry point for other community-directed health interventions [29–31]. However only one example could be found where preventative programs targeting human health have been integrated with programs targeting animal health [32]. In the Schelling et al. study (referred hereafter as the Chad Study) it was reported that combining vaccination for nomadic pastoralists and their livestock in to a single delivery program was a popular approach that resulted in reduced operational costs.

The overarching goal of this proof-of-concept study was to demonstrate the feasibility of the concept of integrating community-wide MDA for STH and MDRV for rabies.

The first objective was to determine whether coupling MDA with MDRV was socially acceptable. The second objective was to investigate how integration impacted a) household participation, b) dog vaccination coverage, c) administration and delivery costs and d) travel time to attend clinics. The third objective was to quantify the impact of the study’s MDA strategy on reaching primary school-aged children (whether or not enrolled in school) and other community members.

Our approach was unconventional because it coupled the responsibilities of the Ministries of human and animal health into one program with the shared aim of preventing multiple NTDs, and it allowed families to bring themselves and their dogs for treatment at one location. This integrated perspective is in line with the ‘One Health’ approach to disease control.

## Methods

The study was carried out in the Ngorongoro District, Tanzania inhabited by semi-nomadic Maasai people. The remote area was chosen because MDRV and MDA are carried out in the region as separate programs. The MDA program delivers twice yearly deworming treatment to every primary school and is coordinated by the District Medical Office (DMO). The MDRV program vaccinates dogs annually and is coordinated by the District Veterinary Office. The MDA and MDRV carried out in this study were one cycle of the established MDA and MDRV in the target villages, respectively. The study took place between February and October 2016, however activities were suspended during the rainy season (April – May) to avoid inclement weather from affecting participation.

**Fig. 1:**
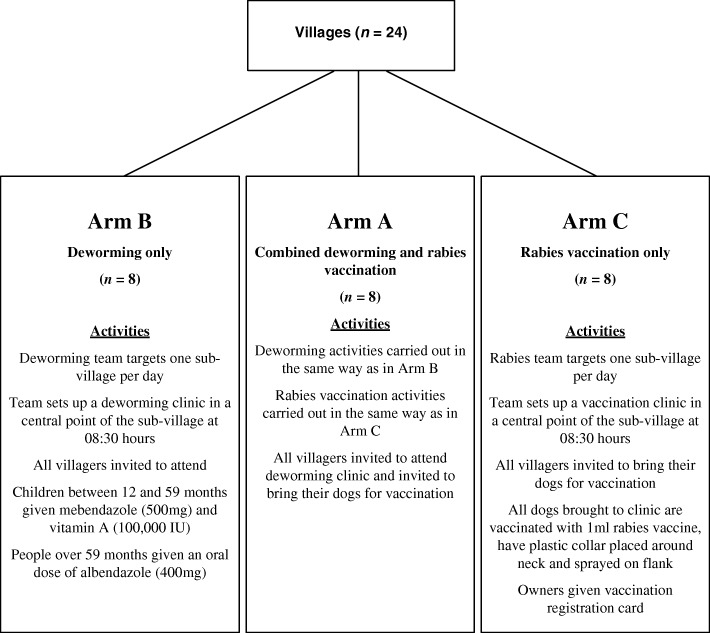
Flow chart describing the distribution of target villages and the activities that took place in each arm of the study

The study focused on 24 villages selected because they are located within the eight wards immediately surrounding the district’s administrative centre. Each of the 24 villages were randomly assigned using a computer randomisation function [33] to one of three arms: i) Arm A (*n =* 8) received both MDA and MDRV; ii) Arm B (*n =* 8) received MDA only; iii) Arm C (*n =* 8) received MDRV only. All villages were equally likely to be assigned to each arm. The distribution of villages between the arms and a summary of activities are shown in Fig. 1. As per the established MDA and MDRV programs, a nurse from the DMO and two village-based community health workers carried out the MDA whilst a rabies field team (a veterinarian, two field staff and a ward-based person) delivered the MRDV. STH and / or rabies awareness information was provided to respondents in the form of verbal disease avoidance advice.

Villages in this region cover a large area, and all are divided into sub-village units. Each intervention (‘event’) was delivered at the level of the sub-village using a ‘central-point’ strategy [34], which required villagers to travel from their homes to the central-point event to receive treatment. Village leaders estimated that hosting each sub-village event for 1 day would provide sufficient time for villagers to attend. Consequently, each event was scheduled to last for 1 day, and the number of days the team(s) spent in each village equalled the number of sub-villages. Because the number of sub-villages ranged between two and six per village, the duration to complete each village was always less than one working week. Consequently, each new village event began on a Monday.

Arm A events comprised an MDA and a MDRV clinic hosted concurrently, while Arm B and Arm C events comprised only one clinic (MDA or MDRV, respectively). For Arm A villages, the MDA and MDRV delivery teams travelled together in one vehicle and set up the clinics close to each other. For Arm B and C villages, the MDA and MDRV teams travelled separately.

In order to quantify the impact of the study’s MDA strategy on reaching primary school-aged children (whether or not enrolled in school) (third objective) the events were all hosted during the school term. In sub-villages with a primary school, the clinics were positioned outside the school grounds, whilst in sub-villages that did not have a primary school the clinics were located in a central location.

To inform each community of the event, a village-wide meeting was convened 1 week before. Key information provided at the meeting included STH and / or rabies awareness information and the importance of controlling these conditions through MDA and MDRV, the date of each clinic, that treatment would be given free of charge, and that people and dogs of all ages were invited to attend. On the Sunday prior to a village event, a motorbike rider with a loudspeaker drove around each village announcing the event details. Additionally, the DMO informed the head teacher of each primary school of the date that the event would be convened outside of the school grounds. At 0830 h the clinic(s) would be set up in the predetermined location and the treatment teams would wait for villagers to arrive. People coming to the MDA clinic were registered and research data collected (see below). Following this, and as stipulated by the standard operating procedure of the program coordinated by the DMO, children between 12 and 59 months were given an oral dose of mebendazole (500 mg) and vitamin A (100,000 IU), people over 59 months were given an oral dose of albendazole (400 mg), and pregnant women were not treated [35]. Following arrival and registration at the MDRV clinics, dogs were vaccinated (Nobivac Rabies®_,_ MSD Animal Health, Boxmeer, Netherlands), a collar placed around the neck and water-soluble purple coloured paint was applied to both flanks. People attending the combined clinic (Arm A) who had also brought dogs were instructed to visit the MDRV clinic *after* receiving deworming treatment. The events ended at 1630 h. Financial administrative costs were collected and used to estimate the cost per treatments under each arm. Economic costs of household participation were collected in the form of travel time and monetized using standard assumptions about time costs.

### Objective 1: community perceptions and knowledge

To gauge opinions about integrated intervention strategies individual interviews (*n* = 59) with village leaders and focus group discussions (FGD, *n* = 30, attempting one men’s and one women’s per village) with a range of participants (range: 8–14, total = 341, mean = 11 / FGD) were carried out.

For the individual interviews, between two and three key informants were selected per village using a combination of purposeful and opportunistic sampling of village leaders (teachers, officials, traditional leaders, etc.). We first approached village government leaders, the Village Chairperson (elected position) or Village Executive Officer (district government appointed position), to determine a participant list of leaders who occupied a pre-determined range of positions of respect (including traditional leaders, teachers, or other members of the village government). Participants from this list were then selected based upon availability. We, therefore, interviewed both those in governmental and traditional leadership capacities (purposeful), which often depended on availability (opportunistic). For the FGD, participants were selected through village leaders and chosen for their leadership roles within, or as respected members of the community.

The interviews were translated from Maasai into English and were analysed using the NVivo® ethnographic software program. Primary analysis was done on the notes of all 30 FGDs, and an initial coding scheme was established. Key theme prevalence across all interviews was assessed after summarizing and shortening responses in an Excel spreadsheet. A selection of representative FGDs (*n* = 13, 7 = women, 6 = men) were purposefully selected for full audio transcription and translation from Maasai or Swahili to English and deeper qualitative analysis. Criteria for selection of FGDs for transcription included clarity of discussion, articulation of ideas and thematic saturation.

### Objective 2a: household participation and dog ownership

To estimate i) the proportion of households that attended the clinics, ii) the proportion of households that kept dogs and iii) the mean number of dogs per household, a post-intervention household questionnaire survey (HQS) targeting randomly selected households (maximum of 30 households per village) was carried out within 1 week of each intervention. A target household list comprising 50 randomly selected households was used to allow for additional households to be selected if households refused to participate or nobody was at home. Because the interventions took a maximum of 6 days per village to complete, and to allow the implementation teams and the HQS data collection team to remain synchronised, the HQS data collection was also designed to last for no more than 6 days. Consequently, approximately five household per day were targeted, with approximately the same number of households being targeted in each sub-village.

Because of budgetary and time limitations, HQS (and coverage estimation) was carried out in only 16 of the 24 target villages. All eight villages in Arm A were targeted, whilst four randomly selected villages in each of Arms B and C were chosen prior to the onset of activities. No households declined to participate, however if the family were not at home the team moved onto the next household on the target household list. Chi squared tests were used to determine whether household participation was impacted by delivery strategy.

### Objective 2b: dog vaccination coverage

A capture-mark-re-sight transect survey method was used to estimate the proportion of dogs that were vaccinated [26]. In brief, a MDRV team member walked through the sub-village on the day after the MDRV clinic and counted the number of dogs, noting whether each had been marked on the flank with the coloured spray (vaccinated) or not (unvaccinated). These sub-village totals were then used to estimate the proportion of dogs for each village that had been vaccinated. This estimation was performed in all eight Arm C villages and six out of the eight Arm A target villages (rainstorms made access difficult in two villages). The proportions of dogs that were vaccinated or not in the villages in each arm were compared using a generalized linear mixed model (binomial family, with village as a random effect).

### Objective 2c: administration and delivery costs

Clinic administration and delivery (A&D) costs were collected for all 24 villages, including variable costs (per dose delivered, by dose type) and fixed costs (per clinic, by clinic type). These costs allowed a cost per dose (A&D) to be calculated. A detailed description of how this data was collected and attributed to each Arm of the study has been given in Additional file 1. In brief, Method 1 calculates the total cost over all clinics (by clinic type and Arm) divided by the total number of doses delivered (by clinic type and Arm) and represents the aggregate cost per dose for a given clinic category. Method 2 calculates cost per dose on a per clinic basis, and then averages over all clinics. Method 1 is useful as an aggregate measure over all clinics but cannot be used to test for statistical differences across clinic categories because it is not calculated on a per clinic basis. Method 2 allows testing for statistical differences across clinic types but represents a summary statistic for clinic-level cost per dose measure rather than an aggregate measure. The exchange rate for cost calculations was 2100 Tsh per U.S. Dollar, approximately the exchange rate that prevailed from mid-2015 through 2016 [36]. Mood’s median and Mann-Whitney Ranksum non-parametric tests were used to test for differences in cost per dose between Arms A and B, and Arms A and C.

### Objective 2d: travel time to attend clinics

To understand which mode of transport was most commonly used, people attending the clinics were asked how they had travelled to the clinic (foot, carried (e.g. infants), bike, car, etc.). To estimate the mean time it took to attend a combined or single event, the same people were also asked how long it took in minutes to reach the events. In addition, the mean amount of time people spent at a clinic and, for the integrated delivery (Arm A) the mean time spent travelling between the two clinics, was measured. These estimates were then used to compare the overall time spent attending single and integrated events. A detailed description of this analysis is given in Additional file 1.

### Objective 3: reaching school-aged children and others

To count the number of participating primary school age children (7–13 years) that were enrolled or not in school, every person (or accompanying guardian if the person was a child) treated in all 16 Arm A and B villages was asked whether they were enrolled in primary school and, if so, which primary school. Every participant was also asked their age, which allowed the range of ages treated to be determined.

To investigate the proportion of enrolled school aged children who received treatment in the MDA events of this study, the number of children registered at the primary schools in the target villages was obtained from the Ngorongoro District NTD Coordinator. To estimate the percentage of enrolled school aged children in each school that received deworming treatment, the number of children enrolled at each school that received treatment was compared to the total number of enrolled children. For reasons unspecified by the NTD Coordinator, registration data was only available for 10 out of the 14 primary schools that participants attended,

All analyses were performed using R [37], except the cost analysis which was performed using Stata® version 14.2. All confidence intervals presented are at the 95% level.

## Results

### Objective 1: community perceptions and knowledge

Analysis of the individual interviews and FGD revealed opinions regarding integration of human / animal health interventions. Opinions of combined human-animal health programs were overwhelmingly positive across all interview platforms. In a yes / no question, 98% of individual interview respondents (*n* = 59) affirmed the combined program was a “good idea” and 88% thought integrating interventions would increase participation. Furthermore, there were positive responses to combining health programs across the FGD with 85% concluding that they resulted in people getting “two for one” health treatments, saved time, effort, and reduced participant costs. However, in the individual interviews, 14% explicitly expressed a negative opinion regarding combined human – animal health interventions, with key negative sentiments being that the strategy could be “unhygienic” or “difficult”. The reasons given most frequently for not participating in health interventions were walking distance (74%), time (61%) and costs associated with attending (17%). In order to gauge which intervention was more highly valued the respondents were asked if they had to choose one intervention, either to have their dogs vaccinated against rabies or their children dewormed, which would they choose? In response, 60% chose to treat their children.

Quotes from respondents when asked to comment on the combined program:

*“To me there are no weaknesses only strengths because from the time when rabies vaccination (program) started we haven’t seen rabid dogs; and also it will help to reduce these problems of worms which have been another problem.”*

*“I see this is good because it reduces cost, we are getting two services at once, and both people and dogs will be treated in the same day.”*

*“Mostly people are willing to participate because of the awareness they have about rabid dogs and how they are dangerous to people; so it’s easy for them to participate willingly at any time when other services are delivered.”*

### Objective 2a: household participation and dog ownership

Full details of all target villages (including village and sub-village names) and the number of dogs vaccinated and / or people that received deworming treatment in each are given in Additional file 2. A summary of the number of people that received deworming treatment and dogs that were vaccinated in each arm follows:

In Arm A eight villages were targeted to receive integrated delivery of MDA and MDRV. In these villages the number of people of all ages that received deworming treatment ranged from 77 to 714 per village and the number of dogs that were vaccinated ranged from 22 to 164 per village. The number of people (within specified age ranges) that were dewormed, and the number of dogs vaccinated in each village and sub-village in Arm A is given in Additional file 3.

In Arm B eight villages were targeted to receive MDA only. In these villages the number of people of all ages that received deworming treatment ranged from 104 to 1014 per village. The number of people (within specified age ranges) that were dewormed in each village and sub-village in Arm B is given in Additional file 4.

In Arm C eight villages were targeted to receive MDRV only. In these villages the number of dogs that were vaccinated ranged from 29 to 141 per village. The number of dogs vaccinated in each village and sub-village in Arm A is given in Additional file 5.

In the villages in which the HQS survey was carried out, up to 30 households per village participated in the HQS. The median percentage of households per village that participated in the MDA clinics in Arm A was 91.5% (range: 22–100%) and in Arm B was 82.5% (range: 69–94%). The median percentage of households per village that participated in the MDRV clinics in Arm A was 86.5% (range: 20–100%) and in Arm C was 90% (range: 75–100%). The number of households that responded that they did or did not participate in the MDA clinic are shown for each village and sub-village in Additional file 6. The number of households that responded that they did or did not participate in the MDRV clinic are shown for each village and sub-village in Additional file 7. In addition, the median proportion of households in each village and in each Arm that participated in the respective clinics are shown.

When the responses from all of the households that participated in the HQS were combined for each arm of the study, 210 out of 257 (81.7%, CI: 76.5–86.0%) Arm A households and 91 out of 115 (79.1% (CI: 70.8–85.6%) Arm B households stated that they participated in the MDA clinics (χ^2^ = 0.2, *p* = 0.66), and 156 out of 194 (80.4%, CI: 74.3–85.4%) Arm A dog owning households and 75 out of 85 (88.2%, CI: 79.7–93.5%) Arm C dog owning households participated in the MDRV clinics (χ^2^ = 2.0, *p =* 0.16). In Arm A, out of 194 dog owning households that were questioned, 150 (77.3%, CI: 70.9–82.6%) attended both the deworming and rabies clinics, 11 (6.3%, CI: 3.2–9.9%) attended deworming only, 6 (2.8%, CI: 1.4–6.6%) attended rabies only, whilst 27 (13.9% (CI: 9.7–19.5%) attended neither.

Of the 260 households that were asked about dog ownership, 202 (78% (CI:72–82.3%)) owned one or more dogs, and the mean number of dogs per household was 1.9 (CI: 1.7–2.1).

### Objective 2b: dog vaccination coverage

The number of dogs that were counted as being marked and unmarked in each village is given in Additional file 8. The mean proportion of dogs in Arm A and C that were vaccinated was 63.8 and 67.3% (O.R. = 1.2, (95% C.I = 0.7–2.1), z = 0.74, *p* = 0.45), respectively.

### Objective 2c: administration and delivery costs

Clinic administration and delivery (A&D) costs collected for all 24 villages, including variable costs (per dose delivered, by dose type) and fixed costs (per clinic, by clinic type), are given in Table 1, whilst Table 2 provides a summary of the results of the A&D cost analyses. Cost per dose was lower under integrated delivery based on both Method 1 and 2 calculations. Using calculation Method 1 cost per dose for deworming averaged $0.24 and $0.36 for Arm A and B, respectively, a 33% reduction in cost per dose. Cost per rabies vaccination averaged $4.36 and $5.18 in Arm A and C, respectively, a 16% reduction in cost per dose. Using Method 2, the average per-clinic deworming cost per dose was $0.32 for Arm A and $0.47 for Arm B, a 32% reduction in cost per dose (*t* = − 2.5, df = 51, *p* = 0.017; a two-sample Mood’s median test provides *p* = 0.004, and a Mann-Whitney Ranksum test provides *p* = 0.0075). The average per clinic rabies vaccination cost per dose was $5.40 for Arm A and $6.06 for Arm C, an 11% reduction in cost per dose (*t* = − 1.0, df = 56, *p* = 0.31; a two-sample Mood’s median test provides *p* = 0.43, and a Mann-Whitney Ranksum test provides *p* = 0.245). Thus, the cost per deworming dose was lower in Arm A under integrated delivery, and statistically significantly so at conventional levels based on Method 2, which calculates cost per dose on a per-clinic basis and allows statistical testing. Rabies delivery cost was also lower in Arm A, although not statistically significantly so. Rabies delivery costs tended to be higher in total and per dose than deworming, largely because the number of people required for delivery was higher (per diem costs) and prevailing wages for their expertise (embodied in Labour costs).

**Table 1:** Fixed and variable costs, by Arm and clinic type

Arm	Arm A			Arm B	Arm C
Clinic	MDA	MDRV	Both	MDA	MDRV
Fixed Costs	3640	8000	11,640	3900	8584
Variable Costs	169	484	653	115	434
Total costs	3808	8484	12,292	4015	9018

*“Fixed costs” include* i) *event-level* fixed *costs not* attributable *to clinics*, which were allocated equally across all events, *and* ii) event-level fixed costs attributable to either *clinic* type (MDA or MDRV), which were attributed equally across all rabies or deworming events, respectively*.* Because there were two clinics for each Arm A event, the *event-level* fixed *costs not* attributable *to clinics* were *allocated equally across* both *clinics in Arm A* and were therefore half that allocated to Arm B or C*. Variable costs* per clinic were *dose-specific costs* multiplied by the number of doses delivered*.* Costs in 2016 US dollars

**Table 2:** The results of the administration and delivery cost analyses

Method	Cost per dose		Cost per vaccination		Test
	Arm A	Arm B	Arm A	Arm C	
Method 1	$0.24	$0.36	–	–	–
Method 1	–	–	$4.55	$5.40	*–*
Method 2	$0.32	$0.47	–	–	*t* = −2.5, df = 51, *p* = 0.017
Method 2	–	–	$5.65	$6.32	*t* = −1, df = 56, *p* = 0.32

The cost per deworming dose and per rabies vaccination for each Arm of the study as calculated by the two different calculation methods is given. Method 1 has calculated the average of a ratio, whilst Method 2 has calculated the ratio of averages (or totals). Method 1 is useful as an aggregate measure over all clinics, but cannot be used to test for statistical differences across clinic categories because it is not calculated on a per clinic basis. Method 2 allows testing for statistical differences across clinic types, but represents a summary statistic for clinic-level cost per dose measure rather than an aggregate measure. The result of the statistical analysis of Method 2 is given

### Objective 2d: travel time to attend clinics

Across all arms of the study villagers travelled a mean of 22 min to and from the clinics (11 min each way, range 1 to 90 min), almost exclusively on foot, with infants being carried. Time spent at the clinic to register and receive treatment or dog vaccination was approximately 10 min. In Arm A, the walking time between the deworming clinic and the rabies clinic was approximately 1 minute. Therefore, the time taken to attend a non-integrated event (Arm B or C, or Arm A if not bringing dogs) was approximately 32 min per person. The time required for a single person with a dog to attend an integrated event and receive the deworming *and* dog vaccination (Arm A) was 43 min including travel. It thus took 33% less time for a single person and a dog to attend a combined event (e.g. Arm A) than to attend two separate events (Arms B and C).

### Objective 3: reaching school children and others

The proportion of children treated by this study in each of the ten primary schools investigated is given in Table 3. The mean and median percentage reached by this study was 73.6 and 76%, respectively. The proportion of primary school aged children in Arm A and B villages combined that received treatment and stated that they were not enrolled in school was 37%.

**Table 3:** The target, the number and the proportion of enrolled primary school children reached by the study’s MDA deworming events in ten primary schools

School	Target	Treated	Prop
Enguserosambu	333	329	0.99
Kritalo	511	225	0.44
Maaloni	324	208	0.64
Magaiduru	407	239	0.59
Njoroi	261	189	0.72
Oldonyowas	347	311	0.90
Orkuyeni	84	66	0.79
Ololosokwani	549	433	0.79
Orkiu Juu	379	224	0.59
Loliondo	1302	1198	0.92

The deworming treatment coverage of enrolled primary school children achieved by the study in ten comparison schools. TARGET = the number of school children enrolled in each school; TREATED = the number of school children that received deworming treatment in the study; PROP = the proportion of school children in each school that received treatment

**Fig. 2:**
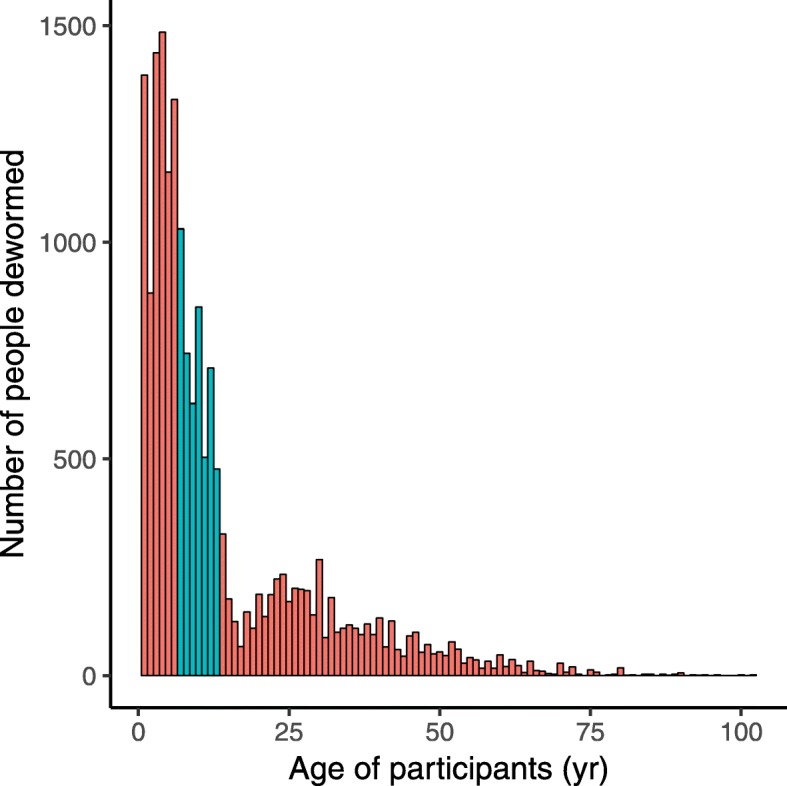
A bar chart showing the number of people of different ages that attended the community-wide deworming events in the study. Blue colour indicates the age range (7–13 years) of primary school aged children in the United Republic of Tanzania that are targeted by the national schools deworming programme (NSDP), whilst the orange colour represents the ages of people treated by the study that would have not been reached by a primary school-based program

Figure 2 shows the age distribution of participants who received deworming treatment and the age range of those eligible to receive treatment through the NSDP. Of people attending the deworming events 21% were primary school age children (blue bars in Fig. 2). Of these, 37% were not enrolled in school. Consequently, 86% of people who came for treatment were not primary school age or were primary school age children not enrolled in school.

## Discussion

In this proof-of-concept study we generated several findings that will be of value in informing the design of integrated delivery interventions to improve human and animal health in hard-to-reach communities. First, we demonstrated that the concept of integrating a MDA focused on human health with one focused on animal health can be feasible and practical. Second, a high proportion of households targeted by the HQS stated that they had participated in the integrated Arm A events, and these proportions were similar to those in Arms B and C where interventions were not integrated. Whilst these figures only represent household participation and not the number of people in each household that attended, these findings, together with the broad community support and the similar rabies vaccination coverages, suggest that coverage might not be reduced when interventions are delivered as part of an integrated delivery strategy. Third, we demonstrated that integrated delivery resulted in a lower cost per dose delivered than if each intervention had been delivered independently. Fourth, because many school-aged children came for treatment who were not enrolled at school, we hypothesise that the strategy employed in this study (in which delivery was located outside of the primary school premises and offered to the whole community) could, in hard-to-reach areas where school enrolment might be relatively low, reach more school-aged children than would be reached through a solely school-based delivery strategy. Fifth, integrated interventions also catalysed effective collaboration between human and animal health workers with establishment of operational inter-sectoral field teams. While this study was carried out in remote areas of Tanzania and the benefits found regarding integrated delivery platforms cannot be generalised to every setting, the insights gained could be broadly applicable to other populations in sub-Saharan Africa that are hard-to-reach and / or underserved by health services.

A limitation of the study was that it was not powered as a full randomized controlled trial. Despite this, steps were taken where possible to randomize selection processes and as such, and despite the small sample size, inferences from the data were drawn. A second limitation was the difficulty obtaining accurate village level human population census data required for the denominator of MDA deworming coverage calculations. As village level census data were not available and we did not have funding to carry out a village-wide census in all target villages, we expected to estimate the human population from the number of households per village. However, due to sub-village divisions and mergers that have occurred in recent years, village executive officers were unable to quantify with confidence the number of households included within their village boundaries. Because of this we used the HQS, performed in 16 of the 24 villages, to calculate the proportion of households in each village and Arm that participated in the interventions. Although we were unable to calculate MDA coverage estimates, the high level of household participation suggests that people were not discouraged from attending a human health event when it was integrated with one providing treatment for animals. This conclusion is supported by the findings from the Chad Study [32] in which a higher number of people were vaccinated when delivery was integrated with livestock vaccination. In a further comparison with the Chad study, strong support was expressed across the interview platforms for integrated delivery approaches suggesting that One Health approaches are viable and, potentially, cost-effective options for delivering health interventions to remote communities. Support for integrated interventions was articulated primarily in terms of time and cost savings and the popularity of receiving multiple health benefits at once. This focus on time saved was borne out in Arm A in which a single person with a dog saved over a third of their time compared to respondents attending separate events. Given that over three quarters of households in the study kept dogs, with a mean of two dogs per household, the delivery of integrated MDA and MDRV interventions has the potential to benefit a large proportion of households. However, the concerns of a small proportion (14%) of informant interviews in describing animal and human health interventions as ‘unhygienic’ and ‘difficult’ should be noted and might need to be addressed in designing future programs.

One concern regarding integrated One Health interventions is that one intervention might compromise the outcomes for the other (e.g. logistical constraints of trying to bring both young children and dogs to receive treatment) and further understanding of potential trade-offs and the nuances of preferences and attitudes would be of value. Indeed, in response to the survey question that asked: ‘*If you had to choose one, which would you choose: to have your dogs vaccinated against rabies or your children wormed?’,* 60% of respondents chose to treat their children. It is not clear whether this reflects a perception that it is better to been seen to say that you would treat children rather than dogs (even if health outcomes of both interventions benefit children) or a more deliberate choice to protect children against the ongoing but less severe effects of worms rather than the more unlikely but lethal outcomes of rabies infection. Although not significant, the coverage of dogs vaccinated was lower in the integrated arm and a larger study might reveal there to be an impact. However, such impacts could potentially be addressed through judicious timing of events. For example, although for research purposes this study was carried out in school term time, it is the authors’ experience that it is often children who are responsible for bringing dogs to central-point clinics for vaccinations and scheduling integrated events during school holidays might make it easier for families to bring both children and dogs to the events, thus improving vaccination coverage.

Another key finding was that the cost per dose delivered was lower under integrated strategies, with delivery of deworming and rabies vaccination reduced, respectively, by $0.12 (33%) and $0.82 (16%) per dose in comparison with single intervention strategies, and, especially for rabies vaccination, these savings appear not to be at the expense of participation in the disease control program. The reduced costs were primarily due to shared transportation costs in Arm A compared to Arms B and C where deworming and rabies teams travelled separately to their respective events. Further, being able to advertise for deworming and rabies vaccination events within the same announcements resulted in further savings in Arm A. Similar cost savings, resulting from shared information campaigns and vehicles, were reported in the Chad Study [32]. Thus, the economies of scope achieved by combining clinics in joint events manifest as cost savings that can offset some reduction in participation in terms of cost-effectiveness.

The mean percentage of enrolled school children treated by this study (74%) was lower than the national average (90%) reported by the NSDP [17], but was broadly similar to an estimate of the coverage achieved within the remote study region (78%) (calculated from data obtained from the Ngorongoro District NTD Coordinator). This suggests that a change of location (i.e. close to, rather than within, a primary school) might not result in a lower coverage of registered primary school children.

The study also suggested that *non-enrolment* in school was considerably higher than the national average (20% in 2014 [38]) with over a third of primary school aged children that received treatment stating that they were not enrolled in school. Because children who did not come for treatment could not be included in the calculation (whether enrolled or non-enrolled), we cannot consider these results indicative of the precise level of non-enrolment. Furthermore, this level of non-enrolment will not be generalizable across Tanzania with specific constraints likely to apply to children in these pastoral communities [18]. Although non-enrolled children can be invited to attend deworming events hosted within schools [39], the level of non-enrolment recorded does suggest that, in hard-to-reach areas, a purely school-based delivery could miss a large fraction of school-aged children, perhaps from households most vulnerable to the health concerns being targeted.

Importantly, not being enrolled at school was not a barrier to children attending the clinics, with many non-enrolled school age children receiving deworming treatment even when the clinics were located just outside of the local primary school and where non-enrolled children might be expected to be wary about presenting themselves (primary school enrolment between the ages of 7 and 13 is compulsory in Tanzania). Moreover, because all age groups were invited, many other community members attended the events. Indeed, the enthusiasm with which villagers attended the community-wide deworming events, and the tone of the responses from the interview platforms, indicated that the strategy was broadly welcomed and that individuals from the community might be effectively reached through extramural programs.

How important is it to reach a broader section of the community? The principle objective of school-based deworming programs is to alleviate morbidity and improve educational and economic outcomes for children [40]. However, treating primary school age children alone will not bring worm burdens sufficiently close to the breakpoint under which transmission is eliminated [19]. Furthermore, any child missed will remain vulnerable to infection and morbidity. Given our findings, this will be a particular concern in areas such as the Ngorongoro District where school enrolment appears low. Moreover, if local elimination of STH is the goal then, in addition to improvements in hygiene, it has been suggested that community-wide treatment of adults as well as children (enrolled in school or not, and of all ages) will likely be required at high levels of coverage (80–90%) over multi-year programs [19]. Irrespective of the scale of the deworming objectives (to reduce morbidity of children only or the cessation of STH transmission) there are likely to be substantial benefits from reaching more children and a broader section of the population. And, given the household participation rates and the enthusiastic attitudes recorded in this study, integrated delivery platforms could play a role in achieving the required levels. However, a larger study is required to fully evaluate the approach tested in this study.

Beyond the added value that may accrue from complementarities and cost-savings, a noticeable, although unmeasured, finding was the inter-sectoral (veterinary / medical) collaboration that developed as a result of the integrated delivery intervention. With teams from different Ministries working together in the field for the first time to achieve common goals, it was apparent that strong collaborative relationships were developing. The benefits that may accrue from this kind of partnership in disease control needs investigating. The study also supports the premise that the community trust established through the delivery of a health intervention with appreciated beneficial outcomes, in this case the reduction in human rabies through dog vaccination, might facilitate integration of a second intervention, here STH treatment. This may be a critical component when the goal is to achieve broad community coverage for program success (e.g. vaccination, drug treatment). With donor and recipient fatigue a growing concern and questions arising about the cost-effectiveness and sustainability of large-scale disease-specific interventions, this study provides optimism that integrated approaches, including One Health strategies that target both human and animal populations, may provide feasible and cost-effective options for delivering health interventions, particularly in hard-to-reach communities.

## Conclusions

This study demonstrated that integrated health delivery platforms can be feasible, popular, cost saving and time saving. Further, integrating human and animal health interventions does not appear to constrain the reach of dog rabies vaccination. The study also demonstrated that children that were not enrolled in school attended the extra-mural intervention. Such an approach could have merit in remote areas where school enrolment might be low. These results indicate the utility of integrated One Health delivery platforms and suggest an important role in the global campaign to reduce the burden of NTDs, especially in hard-to-reach communities.

## Supplementary information

**Additional file 1.** Detailed description of the methodology and analysis for the travel time to attend clinics and the administration and delivery costs.

**Additional file 2.** A list of target villages, the number of people of different age ranges that received deworming treatment and the number of dogs that were vaccinated against rabies.

**Additional file 3.** Numbers of people that received deworming treatment and dogs that were vaccinated in the eight villages in Arm A.

**Additional file 4.** Number of people that received deworming treatment in the eight villages in Arm B.

**Additional file 5.** Number of dogs that were vaccinated in the eight villages in Arm C.

**Additional file 6.** Household participation in eight Arm A and four Arm B deworming clinics determined from the Household Questionnaire Survey (HQS).

**Additional file 7.** Household participation in eight Arm A and four Arm C mass dog rabies vaccination clinics determined from the Household Questionnaire Survey (HQS).

**Additional file 8.** The results of the mark-re-sight survey of dogs in six Arm A and eight Arm C villages.

## Data Availability

The datasets used and/or analysed during the current study are available from the corresponding author on reasonable request.

## Abbreviations

A&D: Administration and Delivery
DMO: District Medical Office
FAO: Food and Agriculture Organization
FGD: Focus group discussion
HQS: Household Questionnaire Survey
I.U.: International Units
MDA: Mass drug administration
MDRV: Mass dog rabies vaccination
NSDP: National primary school-based deworming program
NTD: Neglected tropical disease
OIE: World Organization for Animal Health
STH: Soil transmitted helminth
Tsh: Tanzanian Shilling
WHO: World Health Organization
