# Supplementary material for: An integrated health delivery platform, targeting soil-transmitted helminths (STH) and canine mediated human rabies, results in cost savings and increased breadth of treatment for STH in remote communities in Tanzania
Source: BMC Public Health. 2019 Oct 28;19:1398. doi: 10.1186/s12889-019-7737-6 (PMC6819457; doi:10.1186/s12889-019-7737-6)
Supplement: Supplementary file 1 — Additional file 1. Detailed description of the methodology and analysis for the travel time to attend clinics and the administration and delivery costs. [file 12889_2019_7737_MOESM1_ESM.docx]

## Additional file 1: Travel time to attend clinics

To estimate the mean time ($\bar{t}_{1}$) it took to attend a combined or single event, the same people were also asked how long it took in minutes to reach the events. In addition, the mean amount of time people spent at a clinic ($\bar{t}_{2}$) and, for the integrated delivery (Arm A) the mean time spent travelling between the two clinics ($\bar{t}_{3}$), was measured. These estimates were then used to compare the overall time spent attending single and integrated events. To estimate the overall time a respondent spent attending a single clinic (Arm B or C, or Arm A if no dog was brought for vaccination) $(T_{s})$ we multiplied the mean travel time by two (for the out and return journey) and added the mean time spent at a clinic:

$$T_{s}=\left( \bar{t}_{1} \times2 \right)+ \bar{t}_{2}.$$

To estimate the mean time a respondent spent attending a combined clinic (Arm A) $\left( T_{c} \right)$ we multiplied the mean travel time by two and added the mean time spent at a clinic (multiplied by two for deworming and dog vaccination) and the time spent travelling between the two clinics:

$$T_{c}=\left( \bar{t}_{1}\times2 \right)+\left( \bar{t}_{2}\times2 \right)+ \bar{t}_{3}.$$

## Administration and delivery costs

Clinic administration and delivery (A&D) costs were collected for all 24 villages, including variable costs (per dose delivered, by dose type) and fixed costs (per clinic, by clinic type). Expense categories included: Advertising, Equipment, Incentive payments to village leaders, Labour Costs (team salaries and wage labour for local help), Living (lodging) Allowance, Per Diem for the team, Meeting Costs, Vehicle Fuel, Repairs and Service, Communication (telephone credits), and Other. Other included a variety of miscellaneous costs that do not fit under any other category, such as incidental taxi and transportation costs and team health treatment. Costs attributable to research-related activities were not included in A&D costs. Indirect costs (time, foregone opportunities) borne by households to attend the event (described above) were not included in A&D costs. For accounting purposes, fixed costs not attributable to a specific clinic type were allocated equally across all events. Fixed costs attributable to either MDRV or MDA were attributed equally across all rabies or deworming events, respectively. Because there were two clinics for each Arm A event, the unattributable fixed cost per clinic was half that of Arm B and C. Variable costs per clinic were calculated as per-dose purchase costs multiplied by the number of doses delivered. Per-dose purchase costs were $0.021 (44 Tanzanian Shillings [Tsh]) per deworming dose and $0.26 (520 Tsh) per rabies vaccination. The exchange rate for cost calculations was 2,100 Tsh per U.S. Dollar, approximately the exchange rate that prevailed from mid-2015 through 2016 [1].

The average cost per dose (A&D) was calculated in two ways. Method 1 calculates the total cost over all clinics (by clinic type and Arm) divided by the total number of doses delivered (by clinic type and Arm), and represents the aggregate cost per dose for a given clinic category. Method 2 calculates cost per dose on a per clinic basis, and then averages over all clinics. Both methods provide valid measures of cost per dose (A&D), but provide different estimates, because the average of a ratio (Method 2) is not equivalent to the ratio of averages or totals (Method 1) [2]. Method 1 is useful as an aggregate measure over all clinics, but cannot be used to test for statistical differences across clinic categories because it is not calculated on a per clinic basis. Method 2 allows testing for statistical differences across clinic types, but represents a summary statistic for clinic-level cost per dose measure rather than an aggregate measure.

Mood’s median and Mann-Whitney Ranksum non-parametric tests were used to test for differences in cost per dose between Arms A and B, and Arms A and C.

Reference:

[1] Oander Currency Converter. <https://www.oanda.com/currency/converter/>. Accessed 1^st^ November 2018.

[2] Rao TJ. Mean of ratios or ratio of means or both? J Stat Plan Inference 2002;102:129–38.
