# Supplementary material for: An integrated health delivery platform, targeting soil-transmitted helminths (STH) and canine mediated human rabies, results in cost savings and increased breadth of treatment for STH in remote communities in Tanzania
Source: BMC Public Health. 2019 Oct 28;19:1398. doi: 10.1186/s12889-019-7737-6 (PMC6819457; doi:10.1186/s12889-019-7737-6)
Supplement: Supplementary file 3 — Additional file 3. Numbers of people that received deworming treatment and dogs that were vaccinated in the eight villages in Arm A. [file 12889_2019_7737_MOESM3_ESM.docx]

**Additional file 3: *Numbers of people that received deworming treatment and dogs that were vaccinated in the eight villages in Arm A***

| **ARM** | **VILLAGE** | **SUBVILLAGE** | **< 4** | **5 - 6** | **7 - 13** | **> 13** | **TOTALWORMED** | **RABIES.DOSES** |
| --- | --- | --- | --- | --- | --- | --- | --- | --- |
| A | KRITALO | Empopong' | 134 | 63 | 41 | 129 | 367 | 88 |
| A | KRITALO | Olosirwa | 77 | 63 | 20 | 66 | 226 | 33 |
| A | KRITALO | Karkimuru | 168 | 76 | 54 | 128 | 426 | 42 |
| A | KRITALO | Ilmisigiyo | 175 | 81 | 69 | 141 | 466 | 67 |
| A | KRITALO | Naidikidiko 1 | 43 | 77 | 202 | 84 | 406 | 31 |
| A | MAALONI | Lepolosi 1 | 34 | 53 | 113 | 81 | 281 | 25 |
| A | MAALONI | Ndung'orot | 58 | 25 | 12 | 49 | 144 | 25 |
| A | MAALONI | Loshwash/Kipambi 1 | 211 | 92 | 107 | 304 | 714 | 164 |
| A | MAALONI | Sitet 1 | 72 | 22 | 18 | 62 | 174 | 58 |
| A | MAALONI | Endulelei 1 | 51 | 14 | 26 | 41 | 132 | 48 |
| A | NGOBERETI | Emarti | 252 | 70 | 100 | 204 | 626 | 83 |
| A | NGOBERETI | Enabor Soit | 126 | 40 | 62 | 166 | 394 | 49 |
| A | NGOBERETI | Ngobereti | 250 | 84 | 116 | 238 | 688 | 90 |
| A | NJOROI | Olekinuka | 136 | 31 | 35 | 118 | 320 | 80 |
| A | NJOROI | Oltepes 1 | 76 | 36 | 50 | 67 | 229 | 67 |
| A | NJOROI | Olaika | 88 | 27 | 51 | 64 | 230 | 34 |
| A | NJOROI | Oiti 1 | 76 | 61 | 163 | 90 | 390 | 32 |
| A | OLDONYOWAS | Endakirowa | 91 | 44 | 37 | 150 | 322 | 57 |
| A | OLDONYOWAS | Oldonyowas | 98 | 88 | 194 | 129 | 509 | 71 |
| A | OLDONYOWAS | Loloiboni | 87 | 36 | 27 | 133 | 283 | 53 |
| A | OLOLOSOKWANI | Sero 1 | 221 | 57 | 42 | 146 | 466 | 62 |
| A | OLOLOSOKWANI | Ololosokwan 1 | 10 | 16 | 311 | 48 | 385 | 61 |
| A | ORMANIE | Idupa 1 | 117 | 28 | 20 | 82 | 247 | 91 |
| A | ORMANIE | Ormanie 1 | 127 | 81 | 20 | 86 | 314 | 58 |
| A | ORMANIE | Oitti 1 | 54 | 27 | 19 | 46 | 146 | 49 |
| A | SAKALA | Ndipilikwa | 18 | 3 | 3 | 56 | 80 | 55 |
| A | SAKALA | Lekondia | 26 | 30 | 72 | 45 | 173 | 38 |
| A | SAKALA | Kapongoni | 34 | 25 | 40 | 52 | 151 | 22 |
| A | SAKALA | Bwawani | 18 | 36 | 192 | 123 | 369 | 79 |
| A | SAKALA | Njoriet | 60 | 25 | 14 | 59 | 158 | 39 |
| A | SAKALA | Makalasinga | 21 | 9 | 6 | 41 | 77 | 33 |

The number of people (within specified age ranges) that received deworming treatment and the number of dogs vaccinated in each village and sub-village in Arm A.
