# Supplementary material for: An integrated health delivery platform, targeting soil-transmitted helminths (STH) and canine mediated human rabies, results in cost savings and increased breadth of treatment for STH in remote communities in Tanzania
Source: BMC Public Health. 2019 Oct 28;19:1398. doi: 10.1186/s12889-019-7737-6 (PMC6819457; doi:10.1186/s12889-019-7737-6)
Supplement: Supplementary file 4 — Additional file 4. Number of people that received deworming treatment in the eight villages in Arm B. [file 12889_2019_7737_MOESM4_ESM.docx]

**Additional file 4: *Number of people that received deworming treatment in the eight villages in Arm B***

| **ARM** | **VILLAGE** | **SUBVILLAGE** | **< 4** | **5 - 6** | **7 - 13** | **> 13** | **TOTALWORMED** | **RABIES DOSES** |
| --- | --- | --- | --- | --- | --- | --- | --- | --- |
| B | ENGUSEROSAMBU | Lopiriki | 130 | 56 | 66 | 79 | 331 | NA |
| B | ENGUSEROSAMBU | Olasae | 235 | 98 | 157 | 260 | 750 | NA |
| B | ENGUSEROSAMBU | Ndulele | 75 | 39 | 25 | 44 | 183 | NA |
| B | LOLIONDO | Loliondo Magharibi | 12 | 19 | 209 | 88 | 328 | NA |
| B | LOLIONDO | Loliondo Mashariki | 84 | 124 | 544 | 262 | 1014 | NA |
| B | LOPOLUNI | Lopoluni B | 88 | 44 | 36 | 134 | 302 | NA |
| B | LOPOLUNI | Lopoluni A | 98 | 32 | 342 | 100 | 572 | NA |
| B | LOPOLUNI | Olobo | 98 | 26 | 42 | 61 | 227 | NA |
| B | LOPOLUNI | Arkanda | 105 | 26 | 31 | 79 | 241 | NA |
| B | LOSOITO | Mao | 84 | 18 | 8 | 61 | 171 | NA |
| B | LOSOITO | Losoito 1 | 71 | 35 | 125 | 98 | 329 | NA |
| B | LOSOITO | Kuchinja | 37 | 22 | 15 | 30 | 104 | NA |
| B | MAGAIDURU | Magaiduru | 57 | 12 | 58 | 58 | 185 | NA |
| B | MAGAIDURU | Ndereyani | 56 | 27 | 154 | 113 | 350 | NA |
| B | MBUKEN | Osukunwa | 95 | 19 | 42 | 85 | 241 | NA |
| B | MBUKEN | Mbuken | 107 | 21 | 56 | 112 | 296 | NA |
| B | ORKIU JUU | Orkiu Juu | 158 | 55 | 261 | 181 | 655 | NA |
| B | ORKIU JUU | Olosingo | 81 | 29 | 33 | 78 | 221 | NA |
| B | ORKIU JUU | Orokoroi | 97 | 61 | 24 | 95 | 277 | NA |
| B | ORKIU JUU | Ngototo | 38 | 10 | 9 | 49 | 106 | NA |
| B | ORKUYENI | Ngutoto | 80 | 22 | 38 | 76 | 216 | NA |
| B | ORKUYENI | Orkuyeni A | 81 | 69 | 94 | 62 | 306 | NA |

The number of people (within specified age ranges) that received deworming treatment in each village and sub-village in Arm B.
