# Supplementary material for: An integrated health delivery platform, targeting soil-transmitted helminths (STH) and canine mediated human rabies, results in cost savings and increased breadth of treatment for STH in remote communities in Tanzania
Source: BMC Public Health. 2019 Oct 28;19:1398. doi: 10.1186/s12889-019-7737-6 (PMC6819457; doi:10.1186/s12889-019-7737-6)
Supplement: Supplementary file 5 — Additional file 5. Number of dogs that were vaccinated in the eight villages in Arm C. [file 12889_2019_7737_MOESM5_ESM.docx]

**Additional file 5: *Number of dogs that were vaccinated in the eight villages in Arm C***

| **ARM** | **VILLAGE** | **SUBVILLAGE** | **RABIES DOSES** |
| --- | --- | --- | --- |
| C | MONDOROSI | Olchoki 1 | 58 |
| C | MONDOROSI | Enadoshoke | 55 |
| C | MONDOROSI | Lepolosi 2 | 40 |
| C | MONDOROSI | Orkijiji | 46 |
| C | NAAN | Olchurai 1 | 70 |
| C | NAAN | Naan 1 | 32 |
| C | NAAN | Oloshoo | 64 |
| C | NG'ARWA | Embulubuli | 45 |
| C | NG'ARWA | Ng'arwa | 94 |
| C | NG'ARWA | Oloimoronyai | 88 |
| C | OLOIPIRI | Lajang'a | 75 |
| C | OLOIPIRI | Loswash | 59 |
| C | OLOIPIRI | Esero | 84 |
| C | OLOIPIRI | Oloipiri | 38 |
| C | OLOIRIENI / LOINEN | Lemishiri | 31 |
| C | OLOIRIENI / LOINEN | Lorien | 83 |
| C | OLOIRIENI / LOINEN | Lukumay | 48 |
| C | ORKIU CHINI | Olturoto | 29 |
| C | ORKIU CHINI | Kisamis | 31 |
| C | ORKIU CHINI | Oletet | 31 |
| C | SOITSAMBU | Esilalei | 70 |
| C | SOITSAMBU | Soitsambu | 76 |
| C | SOITSAMBU | Orkoroi | 48 |
| C | SUKENYA | Embash 1 | 91 |
| C | SUKENYA | Sukenya Juu 1 | 73 |
| C | SUKENYA | Olokoboi 1 | 141 |
| C | SUKENYA | Orongai | 69 |

The number of dogs vaccinated in each village and sub-village in Arm C.
