# Supplementary material for: An integrated health delivery platform, targeting soil-transmitted helminths (STH) and canine mediated human rabies, results in cost savings and increased breadth of treatment for STH in remote communities in Tanzania
Source: BMC Public Health. 2019 Oct 28;19:1398. doi: 10.1186/s12889-019-7737-6 (PMC6819457; doi:10.1186/s12889-019-7737-6)
Supplement: Supplementary file 6 — Additional file 6. Household participation in eight Arm A and four Arm B deworming clinics determined from the Household Questionnaire Survey (HQS). [file 12889_2019_7737_MOESM6_ESM.docx]

**Additional file 6:** *H****ousehold participation in eight Arm A and four Arm B deworming clinics determined from the Household Questionnaire Survey (HQS)***

| **VILLAGE** | **SUB-VILLAGE** | **ARM** | **NO** | **YES** | **VILLAGE MEDIAN** | **ARM MEDIAN** |
| --- | --- | --- | --- | --- | --- | --- |
| KRITALO | Empopong' | A | 2 | 6 | 0.75 | 0.92 |
|  | Ilmisigiyo | A | 0 | 4 |  |  |
|  | Karkimuru | A | 2 | 6 |  |  |
|  | Naidikidiko | A | 2 | 6 |  |  |
|  | Olosirwa | A | 0 | 2 |  |  |
| MAALONI | Endulelei | A | 2 | 4 | 0.67 |  |
|  | Kipambi/Loswash | A | 4 | 5 |  |  |
|  | Lepolosi | A | 1 | 3 |  |  |
|  | Ndung'orot | A | 4 | 4 |  |  |
|  | Siteti | A | 1 | 2 |  |  |
| NGOBERETI | Emarti | A | 1 | 18 | 0.97 |  |
|  | Ngobereti | A | 0 | 10 |  |  |
| NJOROI | Oiti | A | 1 | 9 | 0.95 |  |
|  | Olaika | A | 1 | 9 |  |  |
|  | Olekinuka | A | 0 | 6 |  |  |
|  | Oltepes | A | 0 | 4 |  |  |
| OLDONYOWAS | Endakirowa | A | 1 | 9 | 1 |  |
|  | Loloiboni | A | 0 | 15 |  |  |
|  | Oldonyowas | A | 0 | 13 |  |  |
| OLOLOSOKWANI | Mairouwa A | A | 3 | 2 | 0.22 |  |
|  | Mairouwa B | A | 3 | 1 |  |  |
|  | Ololosokwan | A | 2 | 0 |  |  |
|  | Sero | A | 4 | 1 |  |  |
| ORMANIE | Idupa | A | 0 | 9 | 0.88 |  |
|  | Oiti | A | 6 | 4 |  |  |
|  | Ormanie | A | 1 | 7 |  |  |
| SAKALA | Bwawani | A | 0 | 9 | 0.95 |  |
|  | Kapongoni | A | 0 | 6 |  |  |
|  | Lekondya | A | 2 | 6 |  |  |
|  | Makalasinga | A | 2 | 19 |  |  |
|  | Ndipilikwa | A | 2 | 7 |  |  |
|  | Njorwet | A | 0 | 4 |  |  |
| LOPOLUNI | Arkanda | B | 1 | 4 | 0.82 | 0.83 |
|  | Lopolun A | B | 1 | 5 |  |  |
|  | Lopolun B | B | 2 | 5 |  |  |
|  | Olobo | B | 1 | 7 |  |  |
| LOSOITO | Kuchinja | B | 0 | 2 | 0.94 |  |
|  | Losoito | B | 1 | 16 |  |  |
|  | Mao | B | 3 | 6 |  |  |
| MAGAIDURU | Magaiduru | B | 8 | 10 | 0.69 |  |
|  | Ndereyani | B | 2 | 10 |  |  |
| ORKIU JUU | Olosingo | B | 2 | 5 | 0.83 |  |
|  | Orkiu juu | B | 1 | 11 |  |  |
|  | Orokoroi | B | 2 | 10 |  |  |

All HQS target villages and sub-villages in which a deworming clinic was hosted are shown. The number of households targeted by the HQS in each village and sub-village that stated that they did (“YES”) or did not (“NO”) participate in Arm A (combined) or B (deworming only) deworming clinics is also shown. The median proportion of households that participated in the deworming clinic within each village event in Arm A and B (VILLAGE MEDIAN) and the median for each Arm is shown (ARM MEDIAN).
