# Supplementary material for: An integrated health delivery platform, targeting soil-transmitted helminths (STH) and canine mediated human rabies, results in cost savings and increased breadth of treatment for STH in remote communities in Tanzania
Source: BMC Public Health. 2019 Oct 28;19:1398. doi: 10.1186/s12889-019-7737-6 (PMC6819457; doi:10.1186/s12889-019-7737-6)
Supplement: Supplementary file 7 — Additional file 7. Household participation in eight Arm A and four Arm C mass dog rabies vaccination clinics determined from the Household Questionnaire Survey (HQS). [file 12889_2019_7737_MOESM7_ESM.docx]

**Additional file 7: *Household participation in eight Arm A villages and four Arm C villages in mass dog rabies vaccination clinics determined from the Household Questionnaire Survey (HQS)***

| **VILLAGE** | **SUB-VILLAGE** | **ARM** | **NO** | **YES** | **VILLAGE MEDIAN** | **ARM MEDIAN** |
| --- | --- | --- | --- | --- | --- | --- |
| KRITALO | Empopong' | A | 1 | 5 | 0.83 | 0.87 |
|  | Ilmisigiyo | A | 0 | 4 |  |  |
|  | Karkimuru | A | 5 | 3 |  |  |
|  | Naidikidiko | A | 1 | 5 |  |  |
|  | Olosirwa | A | 1 | 1 |  |  |
| MAALONI | Endulelei | A | 1 | 2 | 0.57 |  |
|  | Kipambi/Loswash | A | 3 | 4 |  |  |
|  | Lepolosi | A | 3 | 0 |  |  |
|  | Ndung'orot | A | 3 | 3 |  |  |
|  | Siteti | A | 0 | 2 |  |  |
| NGOBERETI | Emarti | A | 0 | 17 | 1 |  |
|  | Ngobereti | A | 0 | 9 |  |  |
| NJOROI | Oiti | A | 1 | 6 | 0.87 |  |
|  | Olaika | A | 1 | 7 |  |  |
|  | Olekinuka | A | 2 | 4 |  |  |
|  | Oltepes | A | 0 | 2 |  |  |
| OLDONYOWAS | Endakirowa | A | 0 | 7 | 1 |  |
|  | Loloiboni | A | 0 | 8 |  |  |
|  | Oldonyowas | A | 0 | 10 |  |  |
| OLOLOSOKWANI | Mairouwa A | A | 3 | 2 | 0.2 |  |
|  | Mairouwa B | A | 2 | 2 |  |  |
|  | Ololosokwan | A | 2 | 0 |  |  |
|  | Sero | A | 2 | 0 |  |  |
| ORMANIE | Idupa | A | 0 | 6 | 0.86 |  |
|  | Oiti | A | 4 | 2 |  |  |
|  | Ormanie | A | 1 | 6 |  |  |
| SAKALA | Bwawani | A | 0 | 4 | 1 |  |
|  | Kapongoni | A | 0 | 5 |  |  |
|  | Lekondya | A | 1 | 6 |  |  |
|  | Makalasinga | A | 1 | 14 |  |  |
|  | Ndipilikwa | A | 0 | 6 |  |  |
|  | Njorwet | A | 0 | 4 |  |  |
| MONDOROSI | Enadoshoke | C | 1 | 3 | 0.80 | 0.90 |
|  | Lepolosi | C | 3 | 5 |  |  |
|  | Olchoki | C | 1 | 6 |  |  |
|  | Orkijiji | C | 0 | 2 |  |  |
| NAAN | Naan | C | 2 | 2 | 0.75 |  |
|  | Olchurai | C | 1 | 5 |  |  |
|  | Oloshoo | C | 2 | 6 |  |  |
| ORKIU CHINI | Kisamis | C | 0 | 7 | 1 |  |
|  | Oletet | C | 0 | 7 |  |  |
|  | Olturoto | C | 0 | 9 |  |  |
| SUKENYA | Embash | C | 0 | 6 | 1 |  |
|  | Olokoboi | C | 0 | 9 |  |  |
|  | Orongai | C | 0 | 6 |  |  |
|  | Sukenya juu | C | 0 | 2 |  |  |

All HQS target villages and sub-villages in which a mass dog rabies vaccination clinic was hosted are shown. The number of households targeted by the HQS in each village and sub-village that stated that they did (“YES”) or did not (“NO”) participate in Arm A (combined) or C (rabies only) vaccination clinics is also shown. The median proportion of households that participated in the vaccination clinic within each village event in Arm A and C (VILLAGE MEDIAN) and the median for each Arm is shown (ARM MEDIAN).
