# Supplementary material for: An integrated health delivery platform, targeting soil-transmitted helminths (STH) and canine mediated human rabies, results in cost savings and increased breadth of treatment for STH in remote communities in Tanzania
Source: BMC Public Health. 2019 Oct 28;19:1398. doi: 10.1186/s12889-019-7737-6 (PMC6819457; doi:10.1186/s12889-019-7737-6)
Supplement: Supplementary file 8 — Additional file 8. The results of the mark-re-sight survey of dogs in six Arm A and eight Arm C villages. [file 12889_2019_7737_MOESM8_ESM.docx]

**Additional file 8: *The results of the mark-re-sight survey of dogs in six Arm A and eight Arm C villages***

| **ARM** | **VILLAGE** | **MARKED** | **UNMARKED** | **PROP** | **TOTAL** |
| --- | --- | --- | --- | --- | --- |
| A | Maaloni | 92 | 42 | 0.69 | 134 |
| A | Ngobereti | 100 | 82 | 0.55 | 182 |
| A | Njoroi | 81 | 38 | 0.68 | 119 |
| A | Oldonyowas | 86 | 53 | 0.62 | 139 |
| A | Ololosokwani | 64 | 44 | 0.59 | 108 |
| A | Sakala | 82 | 35 | 0.70 | 117 |
| C | Mondorosi | 85 | 82 | 0.51 | 167 |
| C | Naan | 86 | 35 | 0.71 | 121 |
| C | Ng'arwa | 167 | 36 | 0.82 | 203 |
| C | Oloipiri | 139 | 105 | 0.57 | 244 |
| C | Olorien/Loinen | 68 | 59 | 0.54 | 127 |
| C | Orkiu chini | 78 | 12 | 0.87 | 90 |
| C | Soitsambu | 47 | 23 | 0.67 | 70 |
| C | Sukenya | 211 | 90 | 0.70 | 301 |

The number of dogs in six Arm A (combined) and eight Arm C (rabies vaccination only) villages that were recorded as having coloured spray paint on their flanks (MARKED = vaccinated) or not (UNMARKED = unvaccinated) and the proportion (PROP) are shown.
